# Comparison of two handgrip dynamometers in older adults before elective surgery

**DOI:** 10.1186/s13741-023-00334-y

**Published:** 2023-08-23

**Authors:** Maricarmen S. Andrade, Macarena P. Honorato, Javiera P. Vargas, María de los Angeles Galvez, Mariana R. Rojas

**Affiliations:** 1 Department of Geriatrics, Clínica Universidad de los Andes, Santiago, Chile; 2 Department of Geriatrics, Clínica Alemana de Santiago, Vitacura, Chile; 3 Department of Anesthesia, Clínica Alemana de Santiago, Vitacura, Chile

**Keywords:** Perioperative care, Hand strength, Muscle strength dynamometer, Aged, Frail elderly

## Abstract

**Background:**

Handgrip strength is a useful measurement of muscle strength and has been proposed as a single predictor of postoperative outcomes in older adults. The aim of this study was to assess the correlation and concordance of Camry digital hand grip dynamometer (EH101) with gold standard Jamar® hydraulic handgrip dynamometer in older adults previous to elective surgery.

**Methods:**

A cross-sectional study was conducted on patients ≥ 65 years old admitted to a Chilean private hospital for elective surgery between March 2018 and February 2019. Handgrip strength was assessed 2 times with each hand prior to surgery, using both the Jamar® dynamometer and the Camry digital dynamometer. The highest value of each dynamometer was used for analysis.

**Results:**

We included a total of 220 patients (mean age 73.1 years old ± 6.3). Maximal handgrip strength averaged 26.9 kg ± 9.6 with the Camry dynamometer and 26.9 kg ± 9.7 with the Jamar® dynamometer in the right hand and 25.5 kg ± 9.5 with the Camry dynamometer and 25.7 kg ± 9.2 with the Jamar® dynamometer with the left hand. The difference between both measures did not differ significantly from 0, with Pearson correlation index of 0.95 and Lin’s concordance index of 0.95 (*p* < 0001). The Bland–Altman graphics show that 90% of the measures were inside the confidence limits, without systematic bias.

**Conclusion:**

Camry digital dynamometer is an inexpensive and valid device to measure handgrip strength in older adults previous to elective surgery, compared to the gold standard Jamar® hydraulic handgrip dynamometer.

## Background

Hand grip strength is a useful measure of muscle strength, and it has been proposed as a single biomarker of health status in older adults (Bohannon 2019) due to the association to multiple health outcomes as cognitive function, functional status, morbidity, and mortality (Soysal et al. 2021). Handgrip strength has been studied in a preoperative setting in different types of surgery, and weak muscle strength has shown to be a predictor of postoperative adverse outcomes such as hospital stay (Marano et al. 2022), complications rate (Chen et al. 2020), discharge to healthcare facilities, and mortality (Fountotos et al. 2021).

In clinical practice, grip strength is also often used for the assessment of sarcopenia and frailty in older adults (Choe et al. 2020; Chen et al. 2020; John et al. 2013).

The Jamar® dynamometer (J00105 Lafayette Instrument Company, USA) is a widely used hydraulic type dynamometer that presents a high intra and inter-individual reliability and precision and therefore is considered the gold standard for hand grip strength measurement (Roberts et al. 2011; Bohannon and Schaubert 2005). In spite of this, its high cost may limit its use in health centers with reduced financial resources. The Camry digital handgrip dynamometer (EH101; Zhongshan Camry Electronic Co. Ltd., Zhongshan, China) is a spring type dynamometer that has a cost ten times lower than the Jamar® dynamometer. There is increasing evidence with the Camry dynamometer used to measure muscle strength (LinY et al. 2021; Park et al. 2019; Mendes et al. 2020). However, there is scarce evidence that demonstrates that Camry dynamometer is a reliable device in the geriatric population (Muñoz and Millán 2019; Huang et al. 2022), and to our knowledge no previous studies in a preoperative setting.

We hypothesize that the Camry digital dynamometer is a reliable tool to assess handgrip strength in geriatric patients prior to elective surgery, compared to the Jamar® dynamometer.

The aim of this study is to assess the test–retest reliability of the Camry EH 101 dynamometer compared with the gold standard Jamar® device for handgrip strength in older adults prior to elective surgery.

## Methods

### Subjects

Patients ≥ 65 years old admitted for elective surgery in Clínica Alemana of Santiago, Chile, from March 2018 to February 2019. Patients were excluded if they had hand deformation and pain that prohibited the correct use of handgrip and were not able to follow instructions because of a confusional state or dementia. All patients were informed of the nature of the measurements before written informed consent was obtained from the patient or proxy. This study complied with the guidelines set out in the Declaration of Helsinki and was approved by the Ethics Committee of Clínica Alemana of Santiago; the approval number is 2018 (07).

### Study procedures and measures

Before surgery, grip strength (kilograms) was measured on the right hand and then on the left hand with both dynamometers using the Southampton protocol (Roberts et al. 2011). Calibration of the Jamar® dynamometer was performed according to the guidelines set by the manufacturer. The Camry dynamometer was newly purchased before performing the study; the manufacturer advises calibration every 18 months after purchase.

To ensure a similar grip length for all patients, the Jamar® dynamometer was used in the second position, and the Camry dynamometer was used in the third position.

Participants were asked to do maximum effort while they were seated on a chair with back support and fixed armrests, feet flat on the floor, and forearm rested on the arm of the chair. Motivation stimulus was performed by the investigator to encourage the patient to make its maximum grip effort. The body posture during measurement was the same for the Jamar® dynamometer and Camry Dynamometer.

Two values were taken in each hand, with both devices. First, we assessed handgrip strength with the Jamar® dynamometer. Between the two values of the same dynamometer, patients had a 2-min rest and a 15-min rest as a washout time before changing to Camry dynamometer. The highest value of each dynamometer was used for analysis. All data were collected in the RedCap System to protect patient’s data privacy.

### Data analyses

The sample size calculation using Lin’s concordance correlation coefficient, given a 95% precision and 2% loss, indicated we needed 177 patients.

The mean difference of the measurements of both instruments was evaluated. For correlations, Pearson’s correlation coefficient was used. Correlations were considered low (*r* < 0.2), moderate (*r* = 0.2–0.5), or high (*r* > 0.5) according to the recommendations of Cohen (1988). Concordance was assessed by Lin’s concordance coefficient correlation, and Bland–Altman plot for visualization of study results was composed (Bland & Altman 1986).

The maximum strength of handgrip decreases with aging, and there is also a difference between genders (Kubota et al. 2012; Mancilla et al. 2016). Therefore a secondary analysis was performed to determine the gender and age association with the Camry measurements using a regression model and differences in grip strength using *t*-test (*p* < 0.05). For this purpose, we divided into 3 age groups: young old (ages 65–74 years old), middle old (75–84 years old), and very old (≥ 85 years old) (Lee et al. 2018; Strandell and Wolf 2019). An independent association was assessed by a regression model adding gender as a factor.

## Results

### Primary analysis

Two hundred twenty participants 65 years and older were included in the study. Baseline characteristics are shown in Table 1.

**Table 1:** Baseline characteristics

Total participants	220	
Age (mean, SD)	73.1	6.3
Sex (*n*, %)		
Male	106	48%
Female	114	52%
BMI (mean, SD)	27.3	9.0
Weight (kg) (mean, SD)	74.3	13.6
Height (cm) (mean, SD)	165	11.1
Calf circumference (mean, SD)		
Right (cm)	33.5	8.2
Left (cm)	33.4	8.2

*n* number of participants, *SD* standard deviation, *BMI* body mass index, *kg* kilograms, *cm* centimeters

**Fig. 1:**
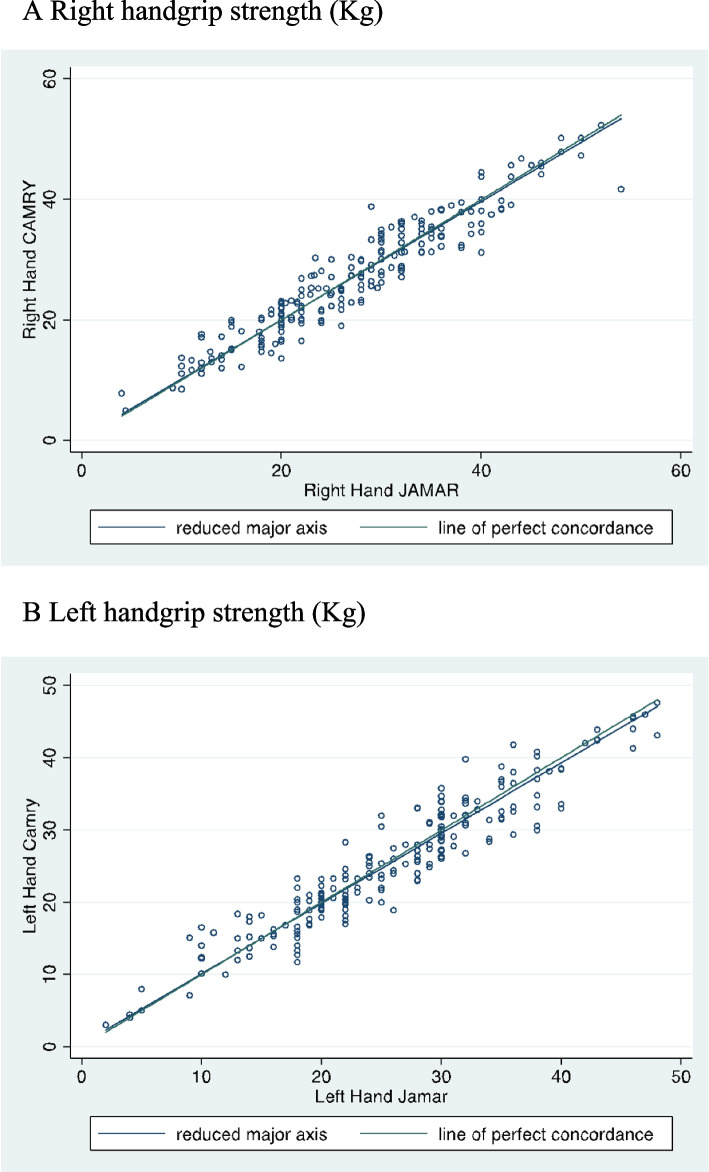
Correlation between two dynamometers in the right hand and left hand. **A** Right handgrip strength (kg). **B** Left handgrip strength (kg)

Grip strength in the right hand was 26.9 kg ± 9.6 with the Camry dynamometer and 26.9 kg ± 9.7 with the Jamar® dynamometer, Pearson correlation coefficient of 0.95 and Lin’s concordance index of 0.95 (scatter plot indicating correlation in Fig. 1A).

Grip strength in the left hand was 25.5 kg ± 9.5 with the Camry dynamometer and 25.7 kg ± 9.2 with the Jamar® dynamometer, Pearson correlation coefficient of 0.95 and Lin’s concordance index of 0.95 (scatter plot indicating correlation in Fig. 1B).

**Fig. 2:**
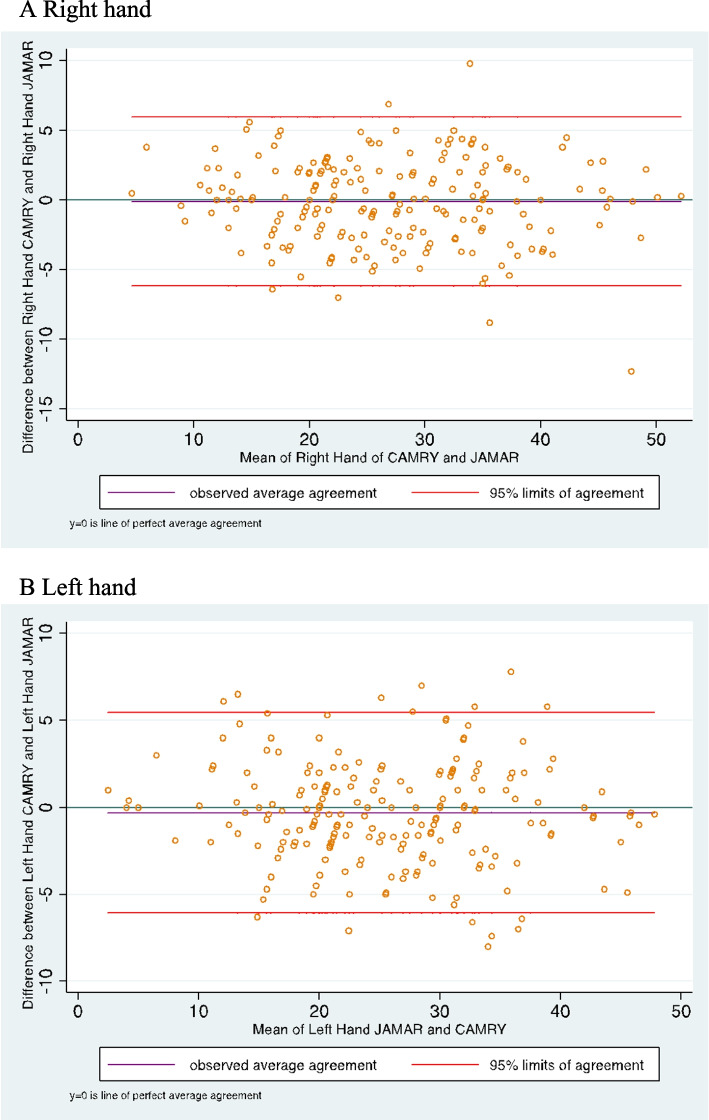
Bland–Altman plot comparing two dynamometers in the right hand and left hand. **A** Right hand. **B** Left hand

The Bland–Altman plot graphics shows that 90% of the measures were between the confidence limits, without systematic bias to greater or smaller measures (Fig. 2A, B). The difference between Camry and Jamar® dynamometer did not differ from 0 in the right hand (difference average =  − 0.11; 95% limits of agreement − 6.2 to + 5.9) and left hand (difference average =  − 0.30; 95% limits of agreement − 6.07 to + 5.45). Both hand differences between Camry and Jamar® have a normal distribution (Shapiro–Wilk test *p* > 0.5).

### Secondary analysis

Grip strength is related to gender (*p* < 0.01) and in women it was statistically less than in men for both hands (*p* < 0.01). Average handgrip measures with Camry Dynamometer for women and men are shown in Table 2.

**Table 2:** Handgrip strength in kilograms (kg) measured with a Camry dynamometer in women and men for the right and left hand

	**Handgrip strength (kg)**
Women (mean, SD)	
Right hand	19.68 ± 0.5
Left hand	19.1 ± 0.6
Men (mean, SD)	
Right hand	33.84 ± 0.7
Left hand	31.5 ± 0.7

When analyzing the grip strength measures with Camry dynamometer in different groups of age, young old (65–74 years old), middle old (75–84 years old), and very old (≥ 85 years old), there is a significant association (*p* < 0.01), and this association is kept when gender (*p* < 0.01) is added to the model. There is a significant statistical difference between the three groups of age (*p* < 0.01). Average handgrip measures by gender and age are shown in Table 3.

**Table 3:** Handgrip strength, measured with a Camry dynamometer in men and women for three groups of ages: young (65–74 years old), middle (75–84 years old), and very old (≥ 85 years old) older adults

	**65–74 (*n* = 142)**		**75–84 (*n* = 59)**		≥ **85 (*n* = 15)**	
	**Men**	**Women**	**Men**	**Women**	**Men**	**Women**
Handgrip strength (mean, SD)						
Right hand (kg)	35.1 ± 7	21.3 ± 5	31.3 ± 6	17.0 ± 5	28.5 ± 7	16.0 ± 5
Left hand (kg)	32.8 ± 6	20.6 ± 5	28.9 ± 7	17.0 ± 6	25.7 ± 1	15.1 ± 6

*n* number of participants, *SD* standard deviation, *kg* kilogram

## Discussion

Our results show that the Camry dynamometer is a valid tool for measuring handgrip strength in older adults before elective surgery, with an excellent agreement that did not differ significantly from zero when compared with the currently gold standard Jamar® dynamometer.

To our knowledge, there are few studies comparing the Camry dynamometer to the gold standard Jamar® dynamometer in older adults, with conflicting results. Huang et al. carried out a study on 1064 healthy community older adults showing excellent reliability and validity when comparing both devices (Huang et al. 2022). However, Diaz Muñoz et al., who studied 133 healthy adults over 18 years old, only showed a good agreement in the age group from 40 to 59 years old; nevertheless, the study only includes 43 older adults (Muñoz and Millán 2019). Handgrip strength might change with different shoulder and elbow positions (Su et al. 1994) without a significant difference between standing and sitting positions (Sousa-Santos and Amaral 2017). This could explain partially the lower average difference obtained in our study compared to the abovementioned ones. In our study, patients were seated with their forearm resting on the arm of the chair using Southampton protocol (LinY et al. 2021) using the same position for both devices. Instead, in Diaz Muñoz et al.’s study, measuring posture was standing with the elbow flexed at 90° for both devices (Muñoz and Millán 2019), and in Huang et al.’s study, measurements with Jamar devices were in a sitting position with a 90° flection using American Society of Hand Therapists protocol and in standing position for Camry dynamometer (Huang et al. 2022). When using a sitting position with the forearm resting over the chair’s arm, it is difficult to use other muscles from the arm and shoulder, and this could give smaller variability between measuring tools.

Previous research shows a variation in grip strength by gender with a higher grip strength in men than in women at all ages and a change associated with age, with a lower grip strength at older age which is concordant with the results of our study (Sousa-Santos and Amaral 2017).

In our secondary analysis, we found an homogenic decline of hand grip strength with age and a difference between women and men, with greater hand grip values for men, which is consistent with previous evidence (Mancilla et al. 2016; Dodds et al. 2016).

Low handgrip strength can be used as a sole predictor of bad outcomes in older adults (Rijk et al. 2016), and in surgery settings, there is evidence that low hand grip strength is associated with increased postoperative morbidity, mortality, and hospital length of stay (Sultan et al. 2012). Having a less expensive device and a valid tool to assess hand grip strength could be broadly used among health centers with different financial resources.

This study has several limitations: the Camry dynamometer we used was newly purchased, and the manufacturer suggests a periodic calibration every 18 months and not in the beginning of its use, so we assumed the device was calibrated. Also, future studies are needed to assess the results after the time of recommended calibration.

There are different recommendations in regard to how many trials should be done to reach maximal handgrip effort and also regarding the rest time between attempts. Most studies suggest 2 to 3 trials with 15 to 60 s of rest between them (Núñez-Cortés et al. 2022). Since the aim of our work was to assess the reliability between two tools, we decided to do 2 attempts to achieve the device’s learning objective of repetition but avoid fatigue. This could have compromised our results since the Camry measurement was the third and fourth (after 2 attempts with Jamar) and could have been systematically better, but there are no biased results in our study given the limits of the agreement are not positively biased. To avoid this issue, a randomized assessment could have been done, but it was not considered in our research, which implies a limitation. Finally, this study was done in an elective preoperative setting, but might be applicable to other settings.

In conclusion, the Camry dynamometer is a valid device to measure hand grip strength in older adults, and it could be considered as an alternative to Jamar gold standard in a preoperative setting and it might be considered in other settings as well. Moreover, being a less expensive device, it could be broadly used among medical centers regarding their budget.

## Data Availability

All data were collected in the RedCap System to protect patients’ data privacy and are available in case they are required.
